# Turning over New Leaves

**Published:** 1888-04-14

**Authors:** 


### Turning over New Leaves.

[Books for Review should be sent to The Editor, The Lodge, Porchester Square, W.]

### Socialist Society.*

The path of the didactic novelist is beset with danger. "Art for art's sake" is a rule to which narrow-minded critics who do not rightly comprehend its meaning may refuse adhesion; but it is one that the artist disobeys at his peril—usually, indeed, to his destruction. In a "story with a purpose," either the story or the purpose must be sacrificed, and as a rule it is the story. There is no help for it. The situations must lead up to the statement of the problem—social, political, or religious—to the elucidation of which the book is devoted. The conversations must always revert to given subjects, and must assume a semi-platonic nature, the neophyte making rash statements and bringing up crude objections for the expert to confute and destroy. Then the characters must act too much on principle, cannot indulge in those vagaries of man and womanhood which form the subject and charm of the average novel; the flexibility, the surprises, and therefore the interest of human life must needs be wanting. Laurence Oliphant has come forth as the romancist of theosophy, and "Masollam" is less perfect as a story, less real as a picture of life, than "Irene Macgillicuddy." The interest of "Lothair," except to those who like it as a crystallised society newspaper, is inferior to that of "Venetia"; nay, all the learning, earnestness, and good intention we meet with in "Daniel Deronda" is not enough to make us enjoy it as we do "Silas Marner." In so far as "Margaret Dunmore," like other novels of its class, is a failure, its author fails in good company. It does not follow that it is an uninteresting book—that is the last thing we should expect from the author of "Scientific Meliorism"; and this story shows the same gifts as that remarkable work—a high and clear strain of thought, and a fearless dealing with social problems. The subject of "Margaret Dunmore" is a sketch in the form of fiction of one of the social improvements suggested in "Scientific Meliorism," the institution of the unitary home, where different families, of all ages, may live together in harmony and mutual help. The notion is on one side a fascinating one, on the other very antagonistic to our English prejudices; but it is worthy of more serious consideration than the "how horrid!" of the unreflecting, or the "it wouldn't work" o the easily sceptical. We English, who boast our attachment to family life, have in our midst a large number of lonely persons—bachelors and spinsters of all ages, and married people whose children are, for one reason or another, removed from their side. All these people lead solitary, and therefore unwholesome lives, forced to seek their interests and enjoyments abroad, and becoming inevitably more and more selfish, and therefore unhappy. They are, for no fault of their own, exiled from that home life we make so much of. Is it not possible for us, by enlarging the boundaries of the family circle, to include them in it? Miss Clapperton thinks it is, and gives us a picture of a household consisting of several young couples, and unmarried and widowed people, young and old. She does not shirk the difficulties and dangers that may result from such combinations, but she believes that in a community whose members are moved by genuinely social feelings these may be got over. And, indeed, we do not see why such a community need fail, if we presuppose this social sentiment, this habit of seeking the good of the community rather than that of the individual. The fact that such households are not common, and that where they have been tried they have resulted in more or less disastrous failure, does not of necessity condemn the principle of the unitary home, but only the individuals composing it. So long as "each man for himself" is the world's rule, and each man and woman seeks to outvie every other, so long will home of this nature be impossible, and exactly so long will homes that have been developed according to natural laws be the abodes of jealous rivalry, and brothers and sisters will be almost vindictively anxious to get on better than each other. But to say that this always must and will be is to deny the ultimate triumph of Christianity, the very foundation of which is altruism. The idea of social obligation has evolved so largely since the days when every man's hand was against every other's, that there is not absolute impossibility in the thought of such a home as Miss Clapperton depicts, where unity and general aim will overcome personal prejudice, and the principle of family love will spring into larger growth. As "Margaret Dunmore" is to some extent a story of the future, we are doing its author no discourtesy in saying that the scheme of the unitary home, attractive as it seems to have been in La Maison, is not at present largely practicable. Though we agree with the dictum that to be free is the only way to learn the use of freedom, we feel that the immediate result of the freedom of the socialist home might be to discredit the whole scheme, unless those who composed it were selected with almost impossible wisdom; but without any avowed, or even conscious intention of carrying out such a scheme, it is surely possible for every existing family to become the nucleus of a larger one, in which the claims of blood and marriage, though not ignored or disparaged, should not be looked upon as the only possible bonds of union. Such a development demands no sharp wrenching asunder of old habits and customs; nothing more than a greater simplicity of manners, and the banishment of the idea that those who are not kin to us are strangers, and therefore in some degree hostile to our interests; and if "Margaret Dunmore" helps to do this, though it may not cause any immediate and startling revolution in society, it will not have been written in vain.

* "Margaret Dunmore; or, A Socialist Home." By J. H. Clapperton, author of "Scientific Meliorism." London: Swan Sonnenschein Lowry, and Co. Price 3*s.* 6*d.*

### Laryngoscopy and Rhinoscopy.*

Specialism in medicine is often spoken against; but if the medical art is to do all that it is capable of doing, and all that the public have a right to expect from it, certain departments of practice must continue to claim the attention of specialists. It is impossible, even for the most Admirable Crichtons of physic, to be perfect in every department of medicine and surgery; and if men of the highest practical capacity cannot compass universal efficiency, what may the commonplace multitude hope to accomplish? It is only by the wise and rightful development of specialism that medicine can make rapid and honest progress. In this little book Dr. Prosser James endeavours to give a series of elementary and progressive lessons to beginners in the speciality of noses and throats. The lessons are models of what such instruction should be. They take it for granted that the learner requires to be taught every part of his business. The writer begins, naturally, with the tools, and then goes on to the method of using them. The lessons are not only conveyed in simple language, but in a way which shows that Dr. Prosser James is able to put himself completely in the pupil's place. The book is meant for students; but every practitioner, although he may not hope or desire to practise the nose and throat specialty, should know enough about noses and throats to be able to examine them fully, and to diagnose at least their ordinary ailments correctly. If he wants to be taught the art of using the rhinoscope and laryngoscope with facility he will not easily find a book better than this for his purpose. As a text-book, both for students and practitioners, it has few equals. The book is small, and though we are not informed, as we ought to be, what the price is, it cannot be high.

* "Laryngoscopy and Rhinoscopy." By Prosser James. London: Bailliere, Tindal, and Cox.

